# Formalin-induced behavioural hypersensitivity and neuronal hyperexcitability are mediated by rapid protein synthesis at the spinal level

**DOI:** 10.1186/1744-8069-5-27

**Published:** 2009-06-07

**Authors:** Curtis O Asante, Victoria C Wallace, Anthony H Dickenson

**Affiliations:** 1 Department of Neuroscience Physiology and Pharmacology, University College London, Gower Street, London WC1E 6BT, UK; 2 Pain Research Group, Department of Anaesthetics, Pain Medicine and Intensive Care, Faculty of Medicine, Imperial College London, Chelsea and Westminster Hospital Campus, 369 Fulham Road, London SW10 9NH, UK

## Abstract

**Background:**

The mammalian target of rapamycin (mTOR) is a key regulator of mRNA translation whose action can be inhibited by the drug rapamycin. Forms of long-term plasticity require protein synthesis and evidence indicates that mRNA in dendrites, axon terminals and cell bodies is essential for long-term synaptic plasticity. Specific to pain, shifts in pain thresholds and responsiveness are an expression of neuronal plasticity and this likely contributes to persistent pain. We investigated this by inhibiting the activity of mTOR with rapamycin at the spinal level, of rats that were subjected to the formalin test, using both behavioural and electrophysiological techniques.

**Results:**

For in vivo electrophysiology, Sprague Dawley rats were fully anaesthetised and single-unit extracellular recordings were obtained from lamina V wide dynamic range (WDR) dorsal horn spinal neurones at the region where input is received from the hind paw. Neuronal responses from naive rats showed that rapamycin-sensitive pathways were important in nociceptive-specific C-fibre mediated transmission onto WDR neurones as well mechanically-evoked responses since rapamycin was effective in attenuating these measures. Formalin solution was injected into the hind paw prior to which, rapamycin or vehicle was applied directly onto the exposed spinal cord. When rapamycin was applied to the spinal cord prior to hind paw formalin injection, there was a significant attenuation of the prolonged second phase of the formalin test, which comprises continuing afferent input to the spinal cord, neuronal hyperexcitability and an activated descending facilitatory drive from the brainstem acting on spinal neurones. In accordance with electrophysiological data, behavioural studies showed that rapamycin attenuated behavioural hypersensitivity elicited by formalin injection into the hind paw.

**Conclusion:**

We conclude that mTOR has a role in maintaining persistent pain states via mRNA translation and thus protein synthesis. We hypothesise that mTOR may be activated by excitatory neurotransmitter release acting on sensory afferent terminals as well as dorsal horn spinal neurones, which may be further amplified by descending facilitatory systems originating from higher centres in the brain.

## Background

The serine-threonine protein kinase mammalian target of rapamycin (mTOR), which is inhibited by the immunosuppressant drug rapamycin regulates several intracellular pathways in response to various extracellular signals, nutrient availability, energy status of the cell and stress. These pathways involve mTOR-dependent activation of the 70 kDa ribosomal protein S6 kinase (p70S6K) as well as the inactivation of the repressor of mRNA translation, eukaryotic initiation factor 4E (eIF4E) binding protein (4EBP) [1,2]. It is therefore not surprising that mTOR activity is modified in a wide range of pathological states such as cancer and neurodegenerative disorders such as Alzheimer's disease [3,4].

Given its widespread implications, it would be logical to hypothesise that rapamycin-sensitive pathways play important roles in persistent pain-like states at the spinal level. Elegant studies investigating the roles of rapamycin-sensitive pathways on injury-induced hyperexcitability of *Aplysia *axons [5]; the roles of local rapamycin-sensitive pathways at the level of the hind paw in a model of nerve injury [6] or the time-restricted roles of rapamycin-sensitive pathways in hippocampal long term potentiation (LTP) [7] reveal insights into the possible roles these mechanisms play in the peripheral and central nervous system. Our studies focus on the spinal mechanisms of pain- an area that like the peripheral mechanisms of pain, generates much interest for many research groups. However, to date, few have investigated the role of spinal protein synthesis pathways in persistent pain-like states.

Kim and colleagues have shown that protein synthesis is an important component of the behavioural hypersensitivity induced by injection of formalin into the hind paw of mice. This was achieved by spinally administering the general transcription inhibitor actinomycin D and the general translation inhibitor anisomycin spinally, prior to formalin injection into the hind paw. The result was an attenuation of behavioural hypersensitivity when compared to spinally administered saline [8]. More recently, Price and colleagues have implicated specific spinal mRNA translation pathways in formalin-induced behavioural hypersensitivity [9]. Their studies focused on mice lacking fragile × mental retardation gene (FMR1), which is another protein that influences mRNA translation. FMR1 is also important for pain processing since it was found that knock out mice displayed reduced formalin-induced behavioural hypersensitivity compared to their wild type littermates. Furthermore, spinal or hind paw administration of rapamycin was ineffective in attenuating formalin-induced behavioural hypersensitivity in the FMR1 mutant mice compared to their wild type littermates showing that not only are rapamycin-sensitive pathways implicated in persistent pain-like states, but that they also interact with other mRNA translation pathways.

The formalin test was first presented by Dubuisson and Dennis in 1977 [10] and is characterised by biphasic ongoing neuronal excitability and behavioural hypersensitivity, which are now commonly used as markers of analgesic drug efficacy [11,12]. We show that rapid mRNA translation mediated by mTOR at the spinal level is necessary for the neuronal hyperexcitability as well as behavioural hypersensitivity induced by formalin that is injected into the hind paw of rats.

## Results

### Rapamycin attenuates baseline neuronal responses under physiological conditions

**Figure 1:**
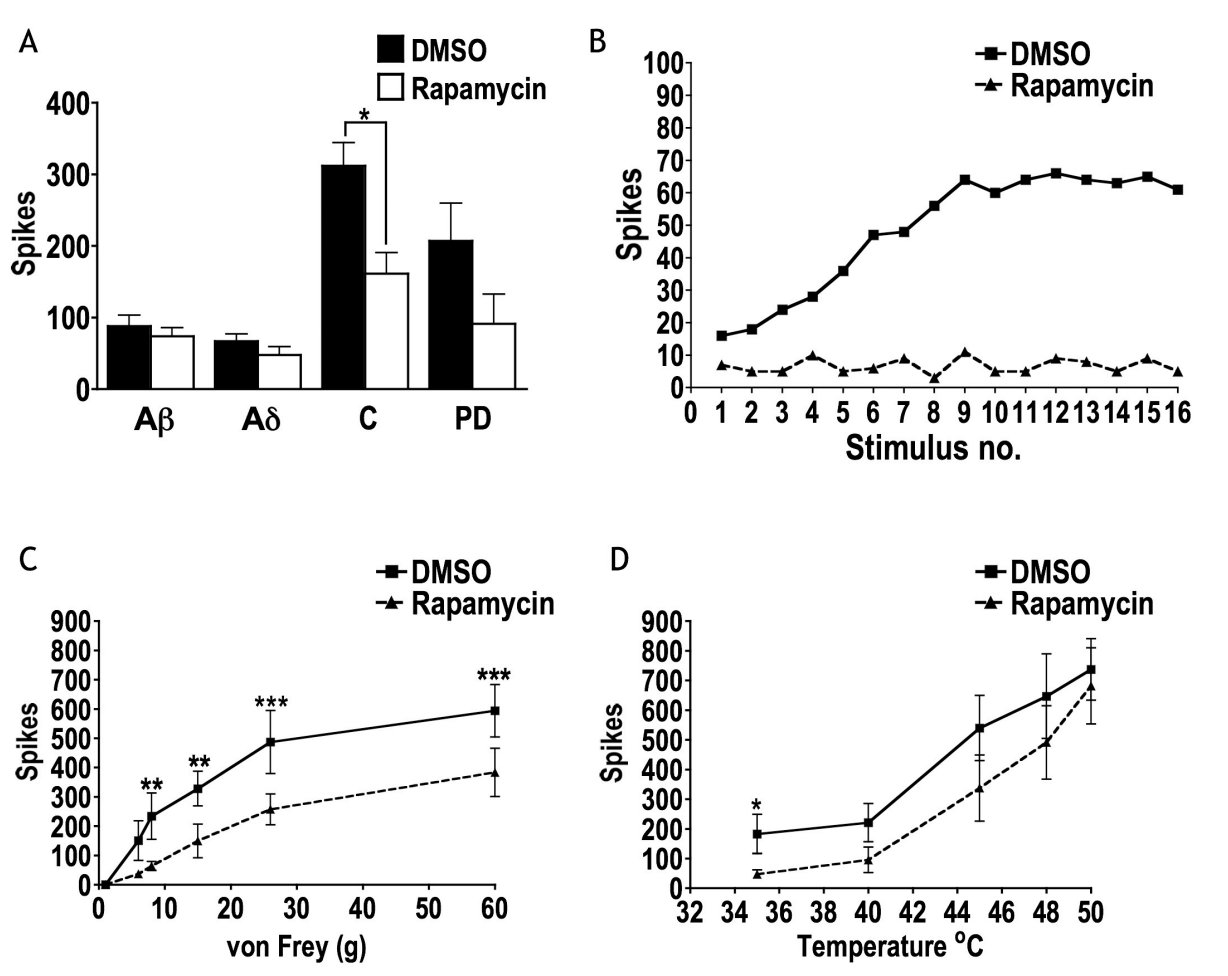
**Effects of spinally administered rapamycin onbaseline neuronal responses**. A. C-fibre mediated transmission onto WDR neurones was significantly inhibited by rapamycin (n = 9) compared to DMSO (n = 10). B. In some cases, there were strong trends for inhibition (as shown in example) by rapamycin of wind up, yet overall, these changes were not significant. C. Rapamycin significantly inhibited responses to 8, 15, 26 and 60 g von Frey filaments when compared to DMSO. D. Rapamycin exerted a significant inhibitory effect on the responses evoked by 35°C. (A. 1 way ANOVA with repeated measures and Dunnett's post-tests. C and D. 2 way ANOVA with repeated measures and Bonferroni's post-tests, *P < 0.05, **P < 0.01, ***P < 0.001).

We used in vivo electrophysiology (see methods) to study the effect of rapamycin on neuronal responses from naive rats in order to determine the importance of rapamycin-sensitive pathways under physiological conditions. When rapamycin was administered onto the exposed spinal cord (250 nM or 11.43 ng in 50 μl), there was a significant reduction in nociceptive-specific C-fibre-mediated transmission onto WDR neurones when compared to DMSO control (Figure 1A), therefore indicating that rapamycin-sensitive pathways mediate stimulus-evoked responses of nocicpetors. We therefore expected that wind-up, which is a potentiated response mediated by nociceptive C-fibre activity and a measure of neuronal hyperexcitability, would also be significantly inhibited. However, this was not case, although in all cases, wind up was inhibited to some extent. Furthermore, in some cases, there were clearly strong inhibitory effects when compared to DMSO (Figure 1B). Although this data my appear conflicting, one must bear in mind that measuring the number of action potentials attributable to C-fibres after a train of stimuli does not directly correlate with wind up since wind up involves the added feature of non linear recruitment of initially silent NMDA receptors [13] which likely requires a higher degree of inhibition achievable, assumingly by a higher dose of rapamycin.

Rapamycin also significantly inhibited neuronal responses to von Frey filaments (8 – 60 g) when compared to DMSO (Figure 1C). Previous studies have shown that in naive rats, the 50% behavioural mechanical withdrawal threshold varies from around 11 to 19 g [14,15] so it is apparent from these results that rapamycin-sensitive pathways are important in mediating neuronal responses to innocuous as well as noxious mechanical stimuli under physiological conditions. Rapamycin-sensitive pathways appear to be more important in mechanically-evoked responses since thermally-evoked responses were weakly altered by rapamycin (Figure 1D). Therefore, although rapamycin-sensitive pathways are important for stimulus-evoked neuronal responses under physiological conditions, this appears to only be true for specific sensory modalities.

### Rapamycin attenuates the second phase of formalin-induced neuronal hyperexcitability

**Figure 2:**
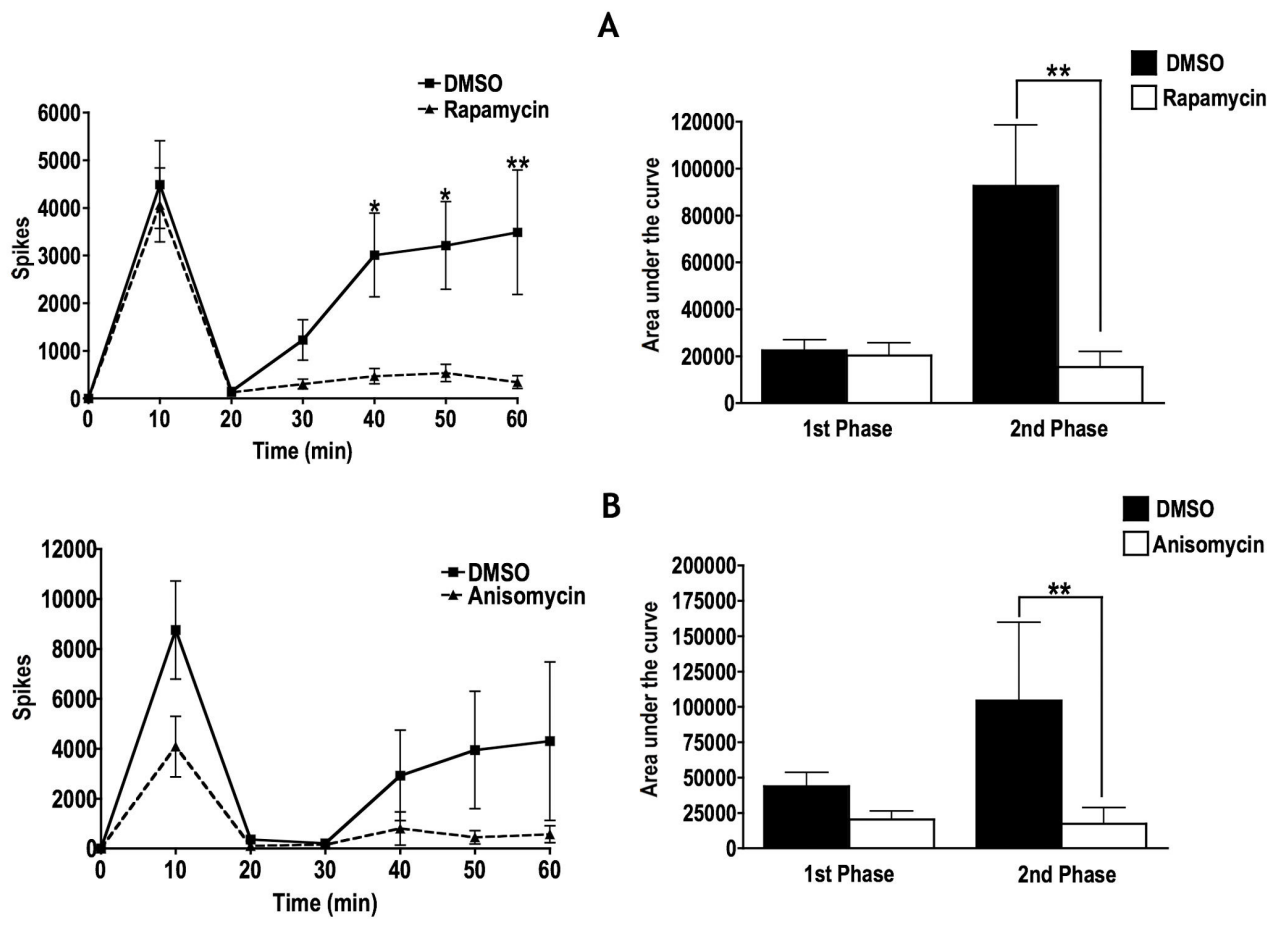
**Effects of spinally administered mRNA translation inhibitors on formalin-induced neuronal hyperexcitability**. A. Pooled data of responses from WDR neurones during the formalin test. When rapamycin (n = 9) was administered onto the exposed spinal cord 3 min prior to 5% formalin injection into the hind paw, there was significant reduction in neuronal activity in the second phase of the test compared to when DMSO (n = 11) was administered to the exposed spinal cord. B. Similarly, when the global translation inhibitor anisomycin (n = 7) was administered onto the exposed spinal cord 3 min prior to formalin injection into the hind paw there was also a significant reduction in neuronal activity in the second phase of the test compared to when DMSO (n = 6) was administered (2 way ANOVA with repeated measures and Bonferroni's post-tests, *P < 0.05; **P < 0.01).

We used in vivo electrophysiology to study spinal neuronal hyperexcitability induced by formalin injection into the hind paw. When rapamycin was administered onto the exposed spinal cord 3 min prior to formalin injection into the hind paw, there was a significant reduction in neuronal activity from 40 – 60 min when compared to DMSO administration. Area under the curve (AUC) analysis confirmed that overall, rapamycin elicited a significant reduction in the second phase of the formalin test (Figure 2A). There were no significant effects of rapamycin on the first phase of the formalin test. To confirm that these effects were indeed due to inhibition of mRNA translation, we also examined the effects of the general mRNA translation inhibitor anisomycin, on the formalin test in the same manner as that for rapamycin. There was a strong trend for inhibition of neuronal hyperexcitability from 10 – 30 min compared to DMSO administration and just like rapamycin, AUC analysis confirmed that overall, anisomycin elicited a significant reduction in the second phase of the formalin test (Figure 2B). A time of 3 min incubation with rapamycin or anisomycin was chosen due to direct evidence from studies on the importance of rapamycin-sensitive pathways in hippocampal LTP pointing towards a time-restricted role for the involvement of rapamycin-sensitive pathways at LTP induction [7]. Spinal neurones selected for 25% DMSO and rapamycin treatment or 10% DMSO and anisomycin treatment prior to formalin being injected into the hind paw comprised equivalent populations for all measures (Tables 1 and 2) i.e. there was no selection bias for neurones for either treatment.

**Table 1:** WDR neurones selected for DMSO or rapamycin treatment prior to hind paw formalin injection comprised equal populations for all measures

	DMSO (n = 11)	Rapamycin (n = 9)
Depth (μM)	777 ± 36	790 ± 51

Aβ-fibre threshold (μA)	0.27 ± 0.10	0.23 ± 0.11

C-fibre threshold (μA)	0.78 ± 0.17	0.55 ± 0.13

Aβ-fibre spikes	104 ± 9	104 ± 16

Aδ-fibre spikes	92 ± 18	93 ± 19

C-fibre spikes	310 ± 49	315 ± 39

Post-discharge spikes	82 ± 29	133 ± 39

Input spikes	298 ± 59	292 ± 57

Wind up spikes	130 ± 33	233 ± 68

35°C spikes	179 ± 52	102 ± 34

40°C spikes	374 ± 91	263 ± 85

45°C spikes	618 ± 67	693 ± 62

48°C spikes	579 ± 104	513 ± 118

50°C spikes	777 ± 91	888 ± 79

All data are expressed as raw mean values ± SEM. There were no significant differences between cells that had the DMSO treatment prior to formalin injection or those that were treated with rapamycin prior to formalin injection (unpaired t-tests, except for graded thermally-evoked responses whereby 2 way ANOVA with Bonferroni's post-tests were used).

**Table 2:** WDR neurones selected for DMSO or anisomycin treatment prior to hind paw formalin injection comprised equal populations for all measures

	DMSO (n = 7)	Anisomycin (n = 6)
Depth (μM)	890 ± 85	771 ± 33

Ab-fibre threshold (μA)	0.70 ± 0.04	0.53 ± 0.11

C-fibre threshold (μA)	1.73 ± 0.24	1.64 ± 0.16

Aβ-fibre spikes	224 ± 40	176 ± 29

Aδ-fibre spikes	189 ± 24	145 ± 26

C-fibre spikes	597 ± 79	432 ± 33

Post-discharge spikes	755 ± 113	549 ± 119

Input spikes	576 ± 112	640 ± 128

Wind up spikes	752 ± 86	486 ± 103

35°C spikes	160 ± 50	261 ± 98

40°C spikes	219 ± 49	351 ± 93

45°C spikes	626 ± 148	544 ± 104

48°C spikes	970 ± 145	904 ± 79

50°C spikes	1409 ± 103	1266 ± 97

All data are expressed as raw mean values ± SEM. There were no significant differences between cells that had the DMSO treatment prior to formalin injection or those that were treated with anisomycin prior to formalin injection (unpaired t-tests, except for graded thermally-evoked responses whereby 2 way ANOVA with Bonferroni's post-tests were used).

### Rapamycin attenuates formalin-induced behavioural hypersensitivity when administered 20 min before formalin

**Figure 3:**
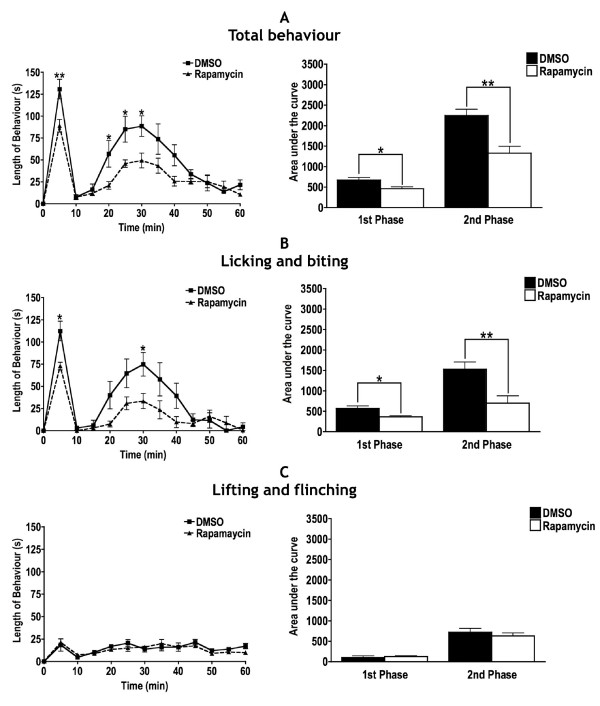
**Effects of spinally administered rapamycin on formalin-induced behavioural hypersensitivity**. A. When rapamycin (n = 6) was administered spinally 20 min prior to 5% formalin injection into the hind paw, there was significant reduction in total behavioural hypersensitivity in both the first and second phase of the formalin test compared to when rats were spinally pre-treated with DMSO (n = 6). B. Specifically, licking and biting behaviour was attenuated when rats were pre-treated with rapamycin. C. Lifting and flinching behaviour however, was unaffected (2 way ANOVA with repeated measures and Bonferroni's post-tests, *P < 0.05, **P < 0.01).

For behavioural studies, we first administered rapamycin 5 min prior to injecting formalin into the hind paw. We found that unlike the results produced with in vivo electrophysiology, there was no significant effect of rapamycin on formalin-induced behavioural hypersensitivity (data not shown). We assume this to be due to the differences in the experimental conditions since the in vivo electrophysiology set up involves applying the drug directly to the exposed spinal cord (dura removed) whereas the behavioural studies involve injecting the drug onto the surface of the cord (dura in tact). In addition, we cannot rule out the possibility that the rats had completely recovered from anaesthesia within 5 min even though they appeared to be fully alert. When rapamycin was spinally administered 20 min prior to formalin injection into the hind paw, there was a significant reduction in the total behaviour for both the first phase at 5 min and also the second phase at 20, 25 and 30 min when compared to DMSO. This was confirmed with AUC analysis (Figure 3A). The effects of rapamycin were found to be more selective for licking and biting as there was a significant reduction in the length of this behaviour in the first phase at 5 min and also in the second phase at 30 min. Again, this was confirmed with AUC analysis (Figure 3B). Rapamycin was however ineffective in attenuating lifting and flinching behaviour (Figure 3C).

## Discussion

These experiments are the first to couple in vivo electrophysiology with behavioural pharmacology during the formalin test to show that rapamycin-sensitive mRNA translation pathways are important in the induction and maintenance of formalin-induced neuronal excitability and behavioural hypersensitivity and therefore may also be important in the induction of clinical persistent pain and even longer lasting chronic pain states.

Using in vivo electrophysiology to study neuronal responses from naive rats, we found that rapamycin significantly inhibited nociceptive-specific C-fibre-mediated transmission onto WDR neurones. This inhibition of C-fibre activity is likely responsible for the accompanying inhibition of mechanically-evoked responses, yet the comparatively minor effects on thermally-evoked responses reveal a selectivity for mechanically-evoked rather than thermally-evoked responses. The fact that rapamycin has an effect on baseline neuronal responses from naive rats suggests that rapamycin-sensitive pathways are at least partially important under physiological conditions. This is perhaps not surprising due to the involvement of mTOR in other physiological processes [4].

Using in vivo electrophysiology, we reveal that formalin-induced neuronal hyperexcitability can be attenuated when rapamycin is administered spinally as early as 3 min prior to formalin injection into the hind paw. In behavioural studies, a lumbar injection of rapamycin 5 min prior to formalin injection did not replicate the results seen with in vivo electrophysiology. However, behavioural hypersensitivity was attenuated when a 20 min pre-treatment period was allowed. This could be due to better access of the drug to its targets in the more static state of in vivo electrophysiology whereby the drug solution is placed directly onto the exposed spinal cord (where the dura is also removed) of the anaesthetised rat or residual effects of the anaesthetic required for the lumbar injection in the behavioural testing.

The first phase of the formalin test is believed to reflect the activity of C-fibre afferent nociceptors, whilst the second phase of the formalin test is believed to be due to central sensitisation of dorsal horn neurones within the spinal cord as a result of the initial barrage of input from C-fibre nociceptor afferents during the first phase [16-18]. Therefore, the finding that rapamycin-sensitive pathways are important in both phases of the formalin test indicates that central spinal rapamycin-sensitive pathways are important in both peripherally driven and centrally-mediated aspects of pain processing.

We also hypothesise a role for higher brain regions in maintaining persistent pain-like states, since rapamycin that inhibits firing of spinal cord WDR neurones to hind paw formalin injection is more effective in reducing licking and biting as opposed to lifting and flinching behaviour. According to optimal scoring strategies, licking and biting has a higher categorical weight than lifting and flinching [19]. It is logical to hypothesise that lifting and flinching behaviour could comprise a significant proportion of reflex behaviour whereas licking and biting may require higher conscious processing (and thus ascending activity from cord to brain through the dorsal horn neurones) to coordinate different muscle groups by the rat with the aim of alleviating the behavioural hypersensitivity. Importantly, the behavioural data confirm that rapamycin-sensitive pathways are important in formalin-induced behavioural hypersensitivity thus correlating with in vivo electrophysiology data where these pathways are important for formalin-induced neuronal hyperexcitability.

Although the importance of spinal rapamycin-sensitive pathways in persistent pain-like states has not been extensively studied, there have been reports on the importance of upstream regulators of mTOR in formalin-induced inflammation. The role of phosphorylated calcium/calmodulin-dependent protein kinase II (CaMKII) and ERK are two such regulators and these proteins have been shown to either engage rapamycin-sensitive pathways or synergise with them, leading to mRNA translation [20-22] and they are upregulated in the dorsal horn of the spinal cord after formalin injection into the hind paw [23]. Also upstream of mTOR is PI3K, which has been recently shown to be important in formalin-induced behavioural hypersensitivity [24] and much like CAMKII and ERK, PI3K has also been shown to engage rapamycin-sensitive pathways [21,22,25-28].

Further upstream, the action of the neurotransmitter glutamate on NMDA receptors has been implicated in formalin-induced neuronal hyperexcitability [29] and is also implicated in activation of rapamycin-sensitive pathways [30,31]. Such is the case with the action of glutamate on the metabotropic glutamate receptors- mGluR1 and mGluR5, which is also of importance in formalin-induced behavioural hypersensitivity [32] as well as activating rapamycin-sensitive pathways [9,33]. Of particular interest here is the finding by Price et al. who showed that mGluR5 antagonism failed to reduce formalin-induced behavioural hypersensitivity in FMR1 mutant mice compared to their wild type littermates, therefore directly showing the engagement of mRNA translation pathways by specific receptor activation as a result of formalin-induced hypersensitivity [9]. Also at the transmitter level, brain-derived neurotrophic factor (BDNF) acting at Trkb receptors has been shown to be important in formalin-induced hypersensitivity [34] and has also separately been shown to activate rapamycin-sensitive pathways [27,28,35,36]. It is therefore clear that a plethora of central neurotransmitters, receptors and subcellular molecules that are important in pain processing likely act via mTOR, implicating rapamycin-sensitive pathways as key mediators of induction as well as maintenance of persistent pain-like states. We hypothesise that not only is mTOR a key regulator of mRNA translation, but that mTOR-dependent mRNA translation is at the root of the neuronal changes and thus the behaviour associated with persistent and chronic pain states.

## Methods

### Animals

For all studies, male Sprague Dawley rats (250 – 280 g) were used. These were supplied by the Biological Services Unit (BSU, University College London, UK). All procedures were carried out in accordance the UK Animals (Scientific Procedures) Act, 1986 and were in agreement with the IASP guidelines [37].

### In vivo electrophysiology

In vivo electrophysiology studies were carried out according to a well established protocol [38]. Rats were initially anaesthetised in an induction box with 4% isofluorane in a mixture of nitrous oxide (66% v/v) and oxygen (33% v/v). Once the rats had lost consciousness and were completely areflexic, the trachea was exposed and isolated and a cannula was inserted into the trachea and fastened with 3-0 silk threads. This was used to maintain anaesthesia throughout the recording period. At this stage, the isofluorane was reduced to 2.5% v/v (areflexia was maintained). Rats were then secured in a stereotaxic frame and a rectal probe attached to a heating blanket was used to maintain a core temperature of 37°C.

An incision was made through the skin along the length of vertebrae and the skin was then separated from the underlying muscle. Muscle, connective tissue and vertebrae were specifically removed from lumbar vertebral segments L1 – L3 of the spinal cord. Muscle and connective tissue from surrounding areas were kept intact and this created a well in the exposed spinal cord area into which, drug solutions could be added. Clamps were used to stabilise and straighten the cord. The dura mater was also removed to aid drug penetration. When the set up was complete, the isofluorane was reduced to 1.8% v/v, a level sufficient for anaesthesia, whilst maintaining areflexia. 11.43 ng rapamycin (sirolimus, LC laboratories) dissolved in a 50 μl saline/dimethyl sulphoxide (DMSO, Sigma) mix comprising 25% v/v DMSO (250 nM rapamycin); 62.35 μg anisomycin (Sigma) dissolved in a in a 50 μl saline/DMSO mix comprising 10% v/v DMSO (4.7 mM anisomycin); 25% v/v DMSO and 10% v/v DMSO were applied directly onto the exposed spinal cord.

Recordings were obtained with an AC recording system (NeuroLog system, Digitimer). An electrode (parylene insulated tungsten microelectrode, 125 μm diameter, 2 MΩ, A-M systems Inc.) inserted into a head stage attached to a 3-axis manipulator was manually lowered into the exposed cord (L4 – L5) to a depth of 500 – 1000 μM. This is an area occupied by WDR neurones that are important in pain processing. An oscilloscope was used to isolate single neurones and a number of stimuli were applied to the receptive field. Mechanical stimuli (von Frey filaments) were applied to the most sensitive part of the receptive field for 10 s. This was also the case for thermal stimuli, where increasing heat was applied using a jet of water from a 60 ml syringe attached to a needle.

Before formalin was administered to the hind paw, a neurone was selected and characterised. Electrical stimuli were delivered by inserting two stimulating electrodes intradermally into the most sensitive part of the receptive field of the hind paw. Firstly, Aβ- and C-fibre thresholds were determined depending on their latencies to respond to stimuli (Aβ-fibres = <20 ms post-stimulus; C-fibres = 90 – 300 ms post-stimulus). The stimulator was then set to three times C-fibre threshold and a train of 16 stimuli (0.5 Hz, 2 ms pulse width) was delivered to the receptive field to determine the number of action potentials attributable to Aβ-fibres (0 – 20 ms); Aδ-fibres (20 – 90 ms); C-fibres (90 – 300 ms) and post-discharge (300 – 800 ms) which is attributable to the wind up elicited by repeated stimuli of nociceptive C-fibres. The input (non-potentiated response) and the wind up (potentiated response) were calculated as follows: C-fibre Input = action potentials (90 – 800 ms) evoked by the first pulse at three times C-fibre threshold multiplied by the total number of pulses (16). This represents the theoretical baseline in the absence of wind up. Wind up = total action potentials (90 – 800 ms) after the 16-train stimulus at three times C-fibre threshold minus the input. This represents the excess activity above the theoretical baseline due to wind up. For characterising thermally-evoked responses prior to hind paw formalin injection, increasing heat was applied using a water jet directed at the receptive field for 10 s. When determining the effect of rapamycin on baseline neuronal responses, only stable cells where 3 consecutive stimulus-evoked responses that were within 10% of the previous result for the same test were selected for further pharmacological study. A 'test' comprising electrical, mechanical and thermal stimuli was carried out every 20 min. Maximum changes (positive or negative) from control in neuronal activity were used for data analysis (all raw values).

To monitor spontaneous neuronal activity as a result of formalin-induced inflammation, 50 μl of a 5% v/v formalin solution made from 40% v/v formaldehyde solution (BDH Chemicals Ltd) was intradermally injected into the hind paw ipsilateral to the WDR neurone which had already been characterised, using a 0.5 ml insulin syringe (BD Micro-Fine™). A WDR neurone was selected on one side of the cord for treatment with spinally administered (intrathecal or i.t.) vehicle prior to formalin injection into the corresponding hind paw. Only after a biphasic control response was achieved was a neurone then selected on the opposite side for treatment with the drug prior to formalin injection into the corresponding hind paw. Neuronal activity was separated into bins of 10 min.

### Behaviour

Before each behavioural study, each rat was allowed to acclimatise for 30 min in individual open top clear Plexiglass chambers (length, width, height = 25 × 25 × 25 cm). In order to determine the effect of drugs at the spinal level on pain-like behaviour, rats were first lightly anaesthetised on 2% v/v isofluorane in a mixture of nitrous oxide (50% v/v) and oxygen (50% v/v) after which they were disinfected and lightly shaved across their backs. A 0.5 ml insulin syringe (BD Micro-Fine™) was used to inject a 20 μl i.t. dose of drug solution (250 μM or 11.43 μg in a 50 μl saline/DMSO mix comprising 25% v/v DMSO) through the skin, into the L5 – L6 vertebral interspace after which, the rats were allowed to recover prior to formalin injection into the hind paw. A 20 μl volume has been shown to produce uniform coverage of the spinal cord which is restricted to the sacral and cauda equina levels and extends up to thoracic T13 – lumbar L1 [39]. Behavioural studies used a higher dose of drug (250 μM) compared with electrophysiological studies (250 nM) since 250 μM rapamycin has been shown to be effective in attenuating capsaicin- and nerve-injury-induced behavioural hypersensitivity when injected locally into the hind paw [6]. After the rats had recovered, they were restrained and 5% v/v formalin solution was then administered to the left hind paw. The rats were then placed back into their chambers and observed for 1 hr. The following behaviours were measured: 1) licking and biting and 2) lifting and flinching [40]. Behavioural data were separated into bins of 5 min. The drug regimen was blinded until the analysis was complete.

After all studies, rats were overdosed on a rising concentration of CO_2_, after which, death was ensured by cervical dislocation of the neck.

## Competing interests

The authors declare that they have no competing interests.

## Authors' contributions

COA conceived, designed and carried out in vivo electrophysiology experiments and behavioural experiments, analysed data and wrote the manuscript. VCW conceived and carried out behavioural experiments. AHD conceived the project and wrote the manuscript.
